# Use of biochemical parameters for non-invasive screening of oesophageal varices in comparison to elastography-based approach in patients with compensated advanced chronic liver disease

**DOI:** 10.11613/BM.2022.020712

**Published:** 2022-06-15

**Authors:** Frane Pastrovic, Anita Madir, Kristian Podrug, Marko Lucijanic, Tomislav Bokun, Marko Zelenika, Sanda Mustapic, Adriana Unic, Lovorka Derek, Ivica Grgurevic

**Affiliations:** 1 Department of Gastroenterology, Hepatology and Clinical Nutrition, University Hospital Dubrava, Zagreb, Croatia; 2 University of Zagreb School of Medicine, Zagreb, Croatia; 3 Department of Gastroenterology and Hepatology, University Hospital Split, Split, Croatia; 4 Department of Hematology, University Hospital Dubrava, Zagreb, Croatia; 5 University of Zagreb Faculty of Pharmacy and Biochemistry, Zagreb, Croatia; 6 Department of Clinical Chemistry, University Hospital Centre Sestre Milosrdnice, Zagreb, Croatia; 7 Clinical Department for Laboratory Diagnostics, University Hospital Dubrava, Zagreb, Croatia

**Keywords:** portal hypertension, cirrhosis, platelet count, esophageal varices, non-invasive tests

## Abstract

**Introduction:**

Oesophageal varices are routinely diagnosed by esophagogastroduodenoscopy (EGD), and their bleeding has high mortality. We aimed to evaluate diagnostic performance of biochemical tests in comparison to elastography-based approaches, as non-invasive alternatives to EGD, for ruling-out high risk oesophageal varices (HRV).

**Material and methods:**

Retrospective analysis of patients (N = 861) who underwent liver stiffness measurement (LSM) by transient elastography (TE) in a single centre over 5-year period, with available results of EGD (within 3 months from LSM). Only patients with suspicion of compensated advanced chronic liver disease (cACLD) defined by LSM ≥ 10 kPa were included comprising the final cohort of 73 subjects. Original and expanded Baveno VI criteria (B6C), controlled attenuation parameter (CAP), platelet count (PLT), aspartate aminotransferase to PLT ratio index (APRI), Fibrosis-4 index (FIB4), model for end stage liver disease (MELD) score were evaluated against the results of EGD that served as the reference method.

**Results:**

Analysed patients had median age 62 years, 59/73 (0.81) were males, 54/73 (0.74) had alcoholic/non-alcoholic fatty liver disease, and 21/73 (0.29) had HRV. In multivariate logistic regression analysis only LSM and PLT were independently associated with HRV. The best performing tests for ruling-out HRV (% of spared EGD; % of missed HRV) were respectively: LSM < 20 kPa (53.4%; 0%), B6C (38%; 0%), Expanded B6C (47.9%; 4.8%); PLT > 214x10^9^/L (21.9%; 0%); FIB4 ≤ 1.8 (21.4%; 0%), APRI ≤ 0.34 (12.3%; 0%). CAP, MELD = 6 alone or combined with PLT > 150(x10^9^/L) did not show acceptable performance.

**Conclusion:**

The best performing biochemical tests for ruling-out HRV in our cohort of patients were PLT and FIB-4, but they were still outperformed by elastography-based approaches.

## Introduction

Portal hypertension (PH) is the key pathophysiologic and prognostic factor in chronic liver disease (CLD), whereas oesophageal varices (EV) indicate the presence of clinically significant portal hypertension (CSPH) (*1*). Moreover, variceal bleeding due to the rupture of EV is a lethal complication of PH, and hence assessing the presence of high-risk varices (HRV) in cirrhotic patients is clinically important to prevent their bleeding (*2*).

Esophagogastroduodenoscopy (EGD) is the gold-standard method for diagnosing EV. However, it is associated with risks due to its invasiveness, and there is also a problem of costs and availability (*3*). The quantitative estimation of PH is possible *via* measurement of the hepatic venous pressure gradient (HVPG), which is also an invasive and expensive method, that requires technical expertise and therefore is limited only to specialized tertiary centers and hence not widely available (*4*, *5*).

Therefore, the possibility to predict the presence of HRV by using a non-invasive test(s) would improve the management of patients with compensated advanced chronic liver disease (cACLD). Currently, a non-invasive blood test, that can predict the severity of portal pressure among patients with cACLD, is not established, although different biochemical tests in combination with other non-invasive tests could be useful in screening patients for the presence of HRV (*6*, *7*). Facing the current COVID-19 pandemics with further limited access to healthcare this approach becomes even more important. Liver stiffness measurement (LSM) by transient elastography (TE) is among the best validated non-invasive methods, with high accuracy for the estimation of the degree of liver fibrosis, and recently has also been applied for the prediction of the presence of CSPH as well as for ruling-out its complications in form of EV in selected cirrhotic patients (*8*). According to the Baveno VI conference (B6C) recommendations platelet count (PLT > 150 x10^9^/L) and LSM < 20 kPa by TE may reliably rule-out HRV in patients with cACLD (*4*). The newer expanded-Baveno VI criteria (EB6C), using PLT count > 110 x10^9^ cells/L and LSM < 25 kPa was demonstrated to spare even more endoscopies than the original criteria with minimal risk of missing HRV in most of the main aetiologies of cACLD (*9*). The risk of missing HRV based on B6C turned out to be less than 5% in most studies. However, in two studies misclassification rate for HRV was > 5% when B6C was used (*10*, *11*).

Controlled attenuation parameter (CAP), a non-invasive method for diagnosing hepatic steatosis, which is also performed along with TE examination, has been demonstrated to correlate with the grade of liver steatosis even in patients with cACLD (*12*). Furthermore, a gradual decrease of the amount of liver steatosis was reported along with the progression of CLD (*13*). However, the performance of CAP has not yet been investigated in terms of diagnosing HRV. Although extensively investigated, reliable and well accepted in clinical practice TE is not universally available and additional non-invasive tools are welcome.

Possible alternative candidates are routine biochemical indices, readily available from the routine blood tests, such as aspartate aminotransferase (AST) to PLT ratio index (APRI), Fibrosis-4 index (FIB4), and Model for end stage liver disease (MELD) score (*14*-*17*).

For the assessment of liver fibrosis in chronic hepatitis C patients, APRI and FIB-4 were initially evaluated (*14*, *15*). The fibrosis-4 index is calculated using the following parameters: age, AST, PLT, and alanine aminotransferase (ALT) (*15*).

Model for end stage liver disease score is a prognostic scoring system, used to predict 3-month mortality in patients with liver cirrhosis (range from 6 to 40; the higher the score, the higher the 3-month mortality related to liver disease). The original MELD score is derived from creatinine and bilirubin concentrations and the international normalised ratio (INR) (*16*).

Although not initially evaluated for this purpose, these biochemical scores were also tested for their performance in diagnosing HRV (*18*, *19*).

In this study, we aimed to investigate the diagnostic performance of biochemical indices: APRI, FIB4, MELD, and PLT+MELD with respect to their ability to ruling-out HRV among the patients with cACLD, as well as to compare them to tests based on elastography such as original B6C, EB6C, and CAP.

## Materials and methods

### Subjects

We retrospectively analysed a cohort of patients who underwent diagnostic work-up due to suspicion of cACLD in the Department of Gastroenterology, Hepatology and Clinical Nutrition of University hospital Dubrava over the 5-year period (1st September 2015 to 1st September 2020). These patients were considered candidates for this study if they had available results of EGD and liver biochemistry parameters performed within 3 months from the date of LSM. Suspicion of cACLD was defined by LSM ≥ 10 kPa as obtained by TE in patients suffering from chronic liver disease without current or previous liver decompensation (EV bleeding, icterus, encephalopathy, or ascites) (*4*). Patients with results of EGD performed > 3 months apart from the date of LSM, with portal vein thrombosis, infiltrative liver neoplasms, and conditions are known for their potential influence on the LSM results (congestive liver disease, extrahepatic biliary obstruction, ALT > 5x Upper limit of normal (ULN)) were excluded (*20*).

**Figure 1:**
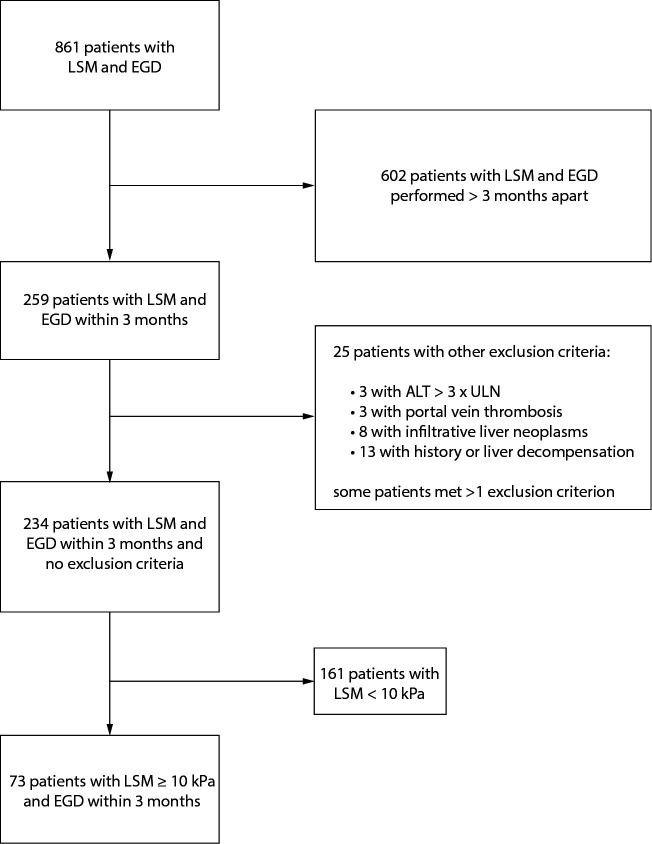
Flowchart of the study. LSM – Liver stiffness measurement (by transient elastography). EGD – esophagogastroduodenoscopy. ALT – alanine aminotransferase. ULN – upper limit of normal.

Eight hundred sixty one patients were identified with available results of LSM by TE, biochemical tests and EGD performed over the investigated period of time. Of them, 602 had EGD performed > 3 months apart from the date of LSM, 25 met other exclusion criteria, and the remaining 234 patients had both LSM results and EGD performed within 3 months from each other. Seventy three out of these 234 patients had the suspicion of cACLD based on LSM ≥ 10 kPa, and thus represented the final cohort eligible for further analysis. The flow chart of the study is depicted in Figure 1.

All included patients had basic demographic, anthropometric, and laboratory results along with LSM and EGD results available from the Institutional information system.

The median age of the 73 patients included in the final analysis was 62 years. A total of 14/73 were females. The most common aetiologies of cACLD were alcoholic liver disease (ALD), in 31/73 patients, and non-alcoholic fatty liver disease (NAFLD) in 23/73 patients. A total of 21/73 patients presented with HRV. Patients’ characteristics stratified according to the presence of HRV are shown in Table 1.

**Table 1:** Patients’ characteristics stratified according to the presence of high risk varices

	**Overall**	**No HRV**	**HRV present**	**P**
Total number	73	52/73 (0.7)	21/73 (0.3)	/
Age (years)	62 (32–77)	63 (32–77)	62 (36–76)	0.976
**Gender**	/	/	/	1.000
Male	59/73 (0.8)	42/52 (0.8)	17/21 (0.8)	/
Female	14/73 (0.2)	10/52 (0.2)	4/21 (0.2)	/
**Aetiology**	/	/	/	Overall 0.397
ALD	31/73 (0.4)	21/52 (0.4)	10/21 (0.5)	0.571
NAFLD	23/73 (0.3)	19/52 (0.4)	4/21 (0.2)	0.145
HBV/HCV	6/73 (0.1)	3/52 (0.1)	3/21 (0.1)	0.345
PBC	6/73 (0.1)	5/52 (0.1)	1/21 (0)	0.666
Other	7/73 (0.1)	4/52 (0.1)	3/21 (0.1)	0.403
**Varices**	/	/	/	Overall < 0.001*
No varices	47/73 (0.6)	47/52 (0.9)	0/21 (0)	< 0.001*
1°	5/73 (0.1)	5/52 (0.1)	0/21 (0)	0.313
2°	19/73 (0.3)	0/52 (0)	19/21 (0.9)	< 0.001*
3°	2/73 (0)	0/52 (0)	2/21 (0.1)	0.024*
SCD (cm)	2.0 (1.8–2.8)	2.0 (1.7–2.5)	2.4 (1.9–3.0)	0.034*
**Probe type** M	39/72 (0.5)	29/51 (0.6)	10/21 (0.5)	0.474
XL	33/72 (0.5)	22/51 (0.4)	11/21 (0.5)	
TE (kPa)	18.3 (13.8–30.3)	14.8 (11.8–19.5)	31.2 (26.3–46.4)	< 0.001*
CAP	290 (245–334)	290 (242–335)	284 (250–328)	0.888
Platelets (x 10^9^/L)	156 (100–206)	173 (107–231)	117 (81–162)	0.019*
PT (%)	87 (71–99)	88 (73–102)	81 (70–88)	0.050*
INR	1.4 (1.3–1.6)	1.4 (1.3–1.5)	1.4 (1.3–1.7)	0.934
Serum creatinine (mmol/L)	69 (62–84)	71 (64–85)	64 (55–76)	0.047*
Bilirubin (mmol/L)	16.5 (11.8–27.2)	15.6 (11.3–22.0)	19.8 (16.0–36.0)	0.031*
Serum sodium (mmol/L)	139 (138–141)	139 (138–141)	139 (137–140)	0.341
AST (U/L)	46 (31–68)	41 (28–63)	51 (36–79)	0.123
ALT (U/L)	40 (27–62)	40 (27–63)	40 (28–58)	0.774
GGT (U/L)	106 (61–273)	103 (61–340)	154 (56–220)	0.807
ALP (U/L)	106 (84–147)	107 (72–145)	105 (88–146)	0.590
APRI score	0.80 (0.49–1.56)	0.70 (0.44–1.11)	1.00 (0.71–2.17)	0.013*
FIB-4 score	2.7 (1.8–5.3)	2.5 (1.5–4.6)	4.4 (2.7–6.2)	0.005*
MELD score	8 (7–11)	8 (7–10)	10 (7–13)	0.153
*Statistically significant at level P < 0.05 (Mann Whintey U test). The results are presented as medians with interquartile range (in parenthesis). ALD – alcoholic liver disease. NAFLD – non-alcoholic fatty liver disease. HBV – hepatitis B virus. HCV – hepatitis C virus. PBC – primary biliary cholangitis. SCD – skin–capsular distance. TE – transient elastography. CAP – controlled attenuation parameter. PT – prothrombin time. INR – international normalized ratio. AST – aspartate aminotransferase. ALT – alanine aminotransferase. GGT – gamma-glutamyl transferase. ALP – alkaline phosphatase. APRI – AST to platelet ratio index. FIB-4 – fibrosis-4 index. MELD – model for end-stage liver disease.				

The study was conducted in accordance with the World Medical Association Declaration of Helsinki and the study protocol was approved by the Ethics committee of the University hospital Dubrava (2020/1012-15).

### Methods

The results of EGD served as the gold standard for diagnosing the presence and grade of EV. Upon the results of EGD grade of EV was classified as follows: grade 0 - no EV; grade 1 - small EV, flattened by the air insufflation; grade 2 - large EV, those protruding into the oesophageal lumen, not flattened upon the air insufflation. EV were considered HRV if they were grade 2 or with cherry red spots (*4*).

Non-invasive approaches to diagnosing HRV including individual biochemical parameters or complex indices (PLT count, APRI, FIB-4, and MELD score), as well as LSM and CAP by TE were evaluated against the results of EGD (Table 1). Results of biochemical analyses were retrieved from the Institutional information system. Only those results that were performed within (+/-) 3 months from the date of TE were considered eligible for the study purpose. This timeframe was related to the date of TE and not EGD because, as already pointed-out, elevated liver aminotransferases might have influenced the diagnostic performance of TE, but not of EGD, and TE was used to define the cohort with suspicion of having cACLD.

Liver stiffness measurement was performed by TE following the international guidelines in the supine position of the patient, after overnight fasting with a right hand in maximal abduction, using the right intercostal approach in a neutral breathing position during the short period of apnoea (*20*). Quality standards of LSM were followed, and only those measurements with the interquartile range (IQR)/median LSM < 30% were considered reliable. Fibroscan M or XL probe was used based upon the suggestion of an automatic probe selection tool incorporated into the Fibroscan Touch 502 vendor (Echosens, France). Quantification of liver steatosis was performed along LSM, and the result was expressed as the median of 10 measurements in dB/MHz.

Due to retrospective design of the study that used only Institutional information system without contacting the patients we had to accept available biochemical results that were recorded in medical files whether they were performed within 3 months before or after TE, as patients were attending their procedures and visits according to the assigned schedule.

Biochemical tests were performed mainly in Clinical department of laboratory diagnostics in University Hospital Dubrava where PLT count was obtained on haematology analyser Advia 2120 (Siemens, Frimley, Camberley, UK), PT/INR on automated haemostasis analysers BCS XP (Siemens, Deerfield, USA) and biochemical parameters on AU2700 plus (Beckman Coulter, Tokyo, Japan) with original manufacturer reagents. In a smaller proportion of patients’ analyses were performed in other certified laboratories in primary care or private clinics as *per* the patients’ convenience. Reference ranges were set up according to Harmonization of Clinical Laboratory Test Results document provided by Croatian Chamber of Medical Biochemists (*21*).

Baveno VI criteria (B6C) for ruling-out HRV were derived from the original consensus document, as follows: PLT count > 150 (x10^9^/L) plus LSM < 20 kPa (*4*).

Expanded Baveno VI (EB6C) criteria were used as described by Augustin S. *et al.*: PLT count > 110 (x10^9^/L) plus LSM < 25 kPa (*22*).

Biochemical scores were calculated based on their respective original formulas, as follows:



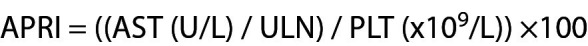





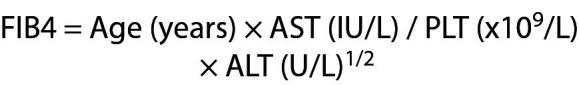





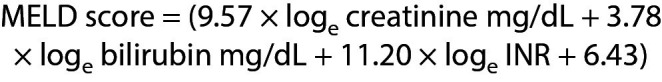



PLT > 150 (x10^9^/L) plus MELD = 6 combination, was the two-step algorithm: first, the patients with PLT > 150 (x10^9^/L) were considered safe to avoid endoscopy, and then patients with PLT < 150 (x10^9^/L) but MELD = 6 were added to them (*17*).

All other combined scores followed the same procedure, *i.e.,* the final number of patients considered as safe to avoid endoscopy was the sum of those who fitted in the first criterion (for example B6C) and the remaining patients outside these criteria but fitting into the second criterion (for example having MELD = 6).

Diagnostic performance of a) tests with already established firm cut-offs: original B6C, EB6C, MELD = 6, PLT > 150 x10^9^/L plus MELD = 6 combinations, b) potentially new and simple tests: controlled attenuation parameter (CAP), c) routine biochemical tests with not well-defined cut-offs for HRV: APRI, FIB4 and PLT count, and d) combinations of the latter with LSM and MELD, were evaluated for their performance in ruling-out HRV.

Patients who fitted within the predefined criteria were considered with a low risk of having HRV and therefore candidates who might have avoided endoscopy. Afterward all patients were analysed for the presence of HRV upon the available results of EGD, and if they had HRV were considered as “missed” HRV. The rate of missed HRV was calculated as the number of patients who indeed had HRV among those classified with low risk according to the tested criteria (*i.e.,* who would otherwise avoid EGD).

### Statistical methods

The normality of the distribution of numerical variables was tested using the Shapiro Wilk test. None of analysed numerical variables had a normal distribution. They were presented as the median and interquartile range (IQR) and were compared between groups using the Mann Whitney U test. Categorical variables were presented as ratios and percentages and were compared between groups using the Χ^2^ test. Age was presented as median and range. Independent associations of different parameters univariately related to the presence of HRV were analysed using logistic regression. All univariately significant variables were included in the model building process *via* backward approach using inclusion criteria P < 0.100 and exclusion criteria P > 0.200. The ROC curve analysis was used to define optimal cut-off levels for the recognition of patients without HRV. Cut-off levels with the highest sensitivity were chosen. An indirect comparison of different criteria for exclusion of HRV was performed without formal statistical testing. Criteria resulting in the highest proportion of spared endoscopies without missing more than 5% of HRV were judged as acceptable. P values < 0.05 were considered to be statistically significant. All analyses were performed using MedCalc statistical software version 19.6 (MedCalc Software Ltd, Ostend, Belgium).

## Results

Patients with HRV were significantly more likely to have larger skin to liver capsule distance, higher LSM by TE, lower PLT count, lower serum creatinine, and higher bilirubin. Patients with HRV also presented with higher APRI and FIB4 scores (P < 0.05 for all analyses, Table 1). Patients with HRV did not differ in age, gender, disease ethology, liver enzymes’ activity (AST, ALT, GGT, ALP), PT or INR, nor MELD score in comparison to non-HRV patients (P > 0.05 for all analyses, Table 1). When analysed in multivariate logistic regression analysis, only TE and platelets (both as continuous variables) remained significantly and independently associated with the presence of HRV (Table 2).

**Table 2:** Logistic regression model for prediction of high risk varices including univariately significant variables

	**OR**	**95%CI for OR**	**P**
TE (kPa)	1.08	1.03–1.14	0.003*
Platelets (x10^9^/L)	0.98	0.96–0.99	0.026*
SCD (cm)	2.49	0.73–8.57	0.146
Serum creatinine (mmol/L)	0.98	0.94–1.01	0.153
FIB-4	0.80	0.61–1.06	0.118
*Statistically significant at level P < 0.05. TE – transient elastography. SCD – skin-capsular distance. FIB-4 – fibrosis-4 index. OR – odds ratio. 95% Cl – 95% confidence interval.			

Diagnostic performance of liver stiffness and biochemistry based scores for prediction of HRV are presented in Table 3. A total of 28/73 and 35/73 patients fulfilled B6C and EB6C, respectively, which also represent the number of potentially spared upper endoscopies that would result in the acceptable rate of missed HRV in 0/28 and 1/35 respectively. The highest safe number of spared endoscopies in comparison to other criteria was achieved by utilizing only LSM < 20 kPa as a criterion, resulting in 39/73 spared endoscopies without missed patients with HRV. On the other hand, PLT count at the established cut-offs (> 150 x10^9^/L for B6C or > 110 x10^9^/L for EB6C) were imprecise with the high number of missed HRV, whereas the cut-off derived from our cohort with 100% NPV was 214 x10^9^/L, and by using this cut-off it would be possible to avoid 16/73 of EGDs with no missed HRV. Controlled attenuation parameter at 100% sensitivity cut-off level ≥ 387 dB/m as derived from our cohort and MELD = 6 did not show meaningful performance. However, using APRI ≤ 0.34 and more so FIB4 ≤ 1.8 (cut-offs with the highest sensitivity, as derived from our cohort) would result in 9/73 and 20/73 of spared EGDs respectively, with no missed HRV. In keeping with the results from logistic regression analysis only marginal improvements in the performance of B6C and EB6C were observed when used in combination with other variables (APRI and FIB4), and even with CAP, whereas combination with MELD = 6 resulted in an unacceptable number of misclassified HRV (4/35 and 4/40, respectively).

**Table 3:** Performance of different clinical criteria for ruling-out high-risk oesophageal varices

**Criteria**	**Sensitivity** **(95% CI)**	**Specificity** **(95% CI)**	**Negative predictive value (95% CI)**	**Spared endoscopies**	**Missed** **HRV**
B6C	100 (84-100)	54 (40-68)	100 (-)	28/73	0/28
EB6C	95 (76-100)	65.4 (50.9-78)	97 (83-100)	35/73	1/35
Platelets > 150 (x10^9^/L)	67 (43-85)	62 (47-75)	82 (71-90)	39/73	7/39
Platelets > 110 (x10^9^/L)	43 (22-66)	73 (59-84)	76 (68-83)	50/73	12/50
Platelets optimal (> 214) (x10^9^/L)	100 (84-100)	30.8 (19-45)	100 (-)	16/73	0/16
LSM < 20 kPa	100 (84-100)	75 (61-86)	100 (-)	39/73	0/39
LSM < 25 kPa	81 (58-95)	83 (70-92)	92 (82-96)	47/73	4/47
LSM optimal (≤ 19.1)	100 (84-100)	73.1 (59-84)	100 (-)	39/73	0/39
CAP optimal (> 387)	100 (83-100)	7.8 (2.2-18.9)	100 (-)	4/72	0/4
MELD = 6 points	81 (58-95)	21 (11-35)	73 (50-89)	15/73	4/15
APRI optimal (≤ 0.34)	100 (84-100)	17 (8-30)	100 (-)	9/73	0/9
FIB4 optimal (≤ 1.8)	100 (84-100)	39 (25-53)	100 (-)	20/73	0/20
B6C + MELD = 6	81 (58-95)	60 (45-73)	89 (76-95)	35/73	4/35
EB6C + MELD = 6	81 (58-95)	69 (55-81)	90 (79-96)	40/73	4/40
Platelets >150 + MELD = 6	57 (34-78.2)	67 (53-80)	80 (70-87)	44/73	9/44
B6B + APRI ≤ 0.34	100 (84-100)	58 (43-71)	100 (-)	30/73	0/30
B6C + FIB4 ≤ 1.8	100 (84-100)	58 (43-71)	100 (-)	30/73	0/30
B6C + CAP > 387	100 (84-100)	62 (47-75)	100 (-)	32/73	0/32
EB6C + APRI ≤ 0.34	95 (76-100)	67 (53-80)	97 (84-100)	36/73	1/36
EB6C + FIB-4 ≤ 1.8	95 (76-100)	67 (53-80)	97 (84-100)	36/73	1/36
EB6C + CAP > 387	95 (76-100)	69 (53-80)	97 (84-100)	37/73	1/37
Gray area – tests with the rate of missed HRV < 5%. LSM – liver stiffness measurement. MELD – model for end stage liver disease. FIB-4 – Fibrosis-4 index. APRI – AST to platelet ratio index. CAP – controlled attenuation parameter. B6C – Baveno VI criteria. EB6C – Expanded Baveno VI criteria. HRV – high-risk varices. LSM – liver stiffness measurement. MELD – model for end stage liver disease.					

A combination of MELD = 6 and PLT > 150 x10^9^/L did not show acceptable performance in our cohort of patients as this algorithm resulted in a high number of missed HRV (9/44).

**Figure 2:**
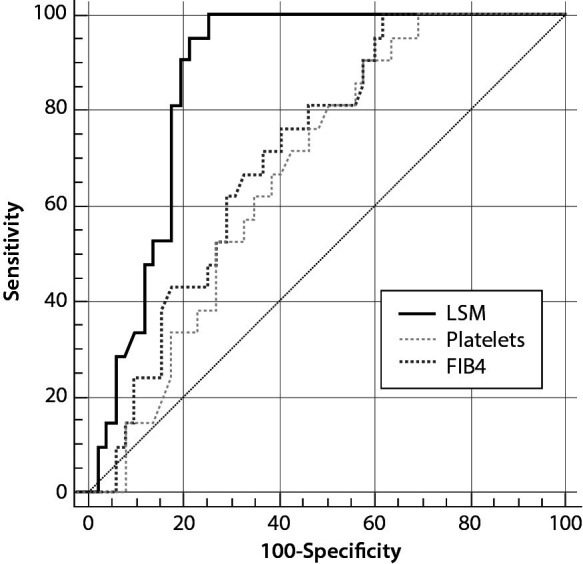
Comparison of ROC curves between FIB4 as the best performing biochemical index and TE and platelets as the components of Baveno VI algorithm for the diagnosis of HRV. LSM – Liver stiffness measurement. FIB-4 – Fibrosis-4 index. TE – transient elastography. HRV – high risk oesophageal varices.

Moreover, area under the curve (AUC) for LSM was 0.87, AUC for platelets was 0.68, and AUC for FIB4 was 0.71 (Figure 2.).

## Discussion

The results of our study show that the highest safe number of spared endoscopies in comparison to other criteria was achieved by utilizing only LSM < 20 kPa as criterion. PLT counts at established cut-offs were imprecise with high number of missed HRV, whereas cut-off of > 214 x10^9^/L resulted in improved performance. Biochemistry based scores APRI and FIB-4 at novel derived cut-off points resulted in lower number of spared endoscopies than those obtained by B6C and EB6C. Controlled attenuation parameter and MELD did not show meaningful performance. Adding biochemistry based scores to B6C and EB6C resulted in only marginal improvement in B6C and EB6C performance.

Among the evaluated indicators LSM by TE and PLT were independently associated with the risk of having HRV. This finding confirms what has been already demonstrated in many studies and finally endorsed by Baveno VI consensus, that combination of LSM by TE and PLT represents reliable and safe non-invasive algorithm for ruling-out HRV (*4*). Although two studies reported misclassification rate of HRV > 5% by using B6C (for patients with chronic hepatitis B) and EB6C (for patients with primary biliary cholangitis), our results are in line with prevailing body of scientific evidence demonstrating that EGD can be safely avoided in 38% of patients by using B6C (*10*, *11*, *23*, *24*). Further on, Expanded B6C have demonstrated even better performance in our cohort with the proportion of patients that might have avoided EGD rising to almost 48%, with slightly increased risk of missing HRV of 4.8%, which is still within the acceptable range of risk as endorsed by Baveno VI consensus. Quoted results from the two studies that failed to demonstrate acceptable performance of B6C/EB6C are probably due to the structure of the investigated cohorts of patients, as it is well known that both hepatitis B and primary biliary cholangitis have different liver stiffness cut-offs when compared to the other aetiologies that prevail (such as NAFLD, ALD or hepatitis C). We also tested CAP, as it was previously demonstrated that decreasing amount of liver fat was observed in patients with more advanced forms of cACLD, and thus lower CAP might also be expected among patients with HRV (*13*). However, CAP did not show meaningful diagnostic performance as it was able to spare only 4/72 of EGDs at very high cut-off > 387 dB/m revealing 100% sensitivity and NPV (but only 7.8% specificity). Addition of CAP > 387 dB/m only marginally improved B6C or EB6C, and thus CAP is not reliable parameter to be used for diagnosing HRV.

Even if TE is safe, reliable, and easy to use, it is still not universally available to patients, so there is still a need for other simple and reliable non-invasive tests. Blood tests represent desirable candidates as they are widely available, standardised and some of them have already demonstrated good diagnostic performance for HRV. Platelets at the published cut-offs > 110 x10^9^/L, and > 150 x10^9^/L (that are used as the part of B6C and EB6C) are rather imprecise when used alone, with a high rate of missed HRV (7/39 and 12/50). This is rather expected based on the previous knowledge, as PLT count might be influenced by various conditions, and not only portal hypertension and therefore PLT count is not a good candidate to be used alone (*25*). Interestingly, both MELD = 6 alone and its combination with PLT > 150 x10^9^/L performed badly in our cohort. This is as opposed to the results from the original study that established MELD = 6 + PLT > 150 x10^9^/L criteria, where the rate of spared EGDs was 54% with 0 missed HRV (*17*). In the validation cohort it was possible to spare 44/73 EGDs, and 9/44 HRV was missed. A potential explanation for such a significant difference between this and the original study might be based on the different structures of the analysed cohorts, as in the original study majority of patients who had hepatitis C (73%), were males (99%), and had very low (9%) prevalence of HRV (*17*).

We also tested the diagnostic performance of APRI and FIB4 for HRV, the well-known scores used for staging liver fibrosis. As both contain PLT count which reflects the presence of PH, with all aforementioned limitations, they might be likely candidates for this purpose. Both tests have already been evaluated for their ability to rule-out HRV with the reported cumulative sensitivities of 0.65 and 0.62, and specificities of 0.66 and 0.64 respectively for APRI and FIB4 in meta-analysis that included 8 studies for APRI and 4 studies for FIB4 (*3*). Reported cut-offs optimized for sensitivity/specificity (Youden index) for APRI ranged from 1.02-2.2 and for FIB4 3.3-7.7. According to our results, when optimised for the highest sensitivity, both tests safely ruled-out HRV with more EGDs spared by using FIB4 ≤ 1.8, but with a significantly smaller proportion of patients fitted into this range as compared to other indices that have been evaluated here. No further improvement of B6C or EB6C performance was observed when APRI or FIB4 were used as an additional criterion, in keeping with the results of multiple logistic regression analysis (Table 2).

This study has limitations due to its retrospective design, recruitment of patients from a single centre and a relatively small number of patients included in the final analysis. On the other hand, presented data reflect a real-life experience outside of defined research protocols, all included patients were compensated with no previous episodes of liver decompensation, had been thoroughly examined and all had EGD and biochemical tests performed within 3 months from the moment of LSM.

In conclusion, the results of this study conducted over the cohort of patients with suspicion of cACLD and no previous decompensation reveal that the best performing non-invasive algorithms for ruling-out HRV are based on LSM. When TE is not available FIB-4 score at cut of 1,8 could be used in order to spare endoscopies and with no risk of missing HRV, according to our results. However larger cohort is needed to confirm our results.

## Data Availability

The data generated and analysed in the presented study are available from the corresponding author on reasonable request.
